# A machine learning approach to behavioral signals of burnout in audit and control professions

**DOI:** 10.3389/fpsyg.2026.1792200

**Published:** 2026-08-27

**Authors:** Ayşe Atılgan Sarıdoğan, Zekeriya Emre Erkal, Nabi Küçükgergerli, Muzaffer Ertürk, Murat Emeç, Adem Yaman

**Affiliations:** 1 Department of Accounting and Tax Applications, Çanakkale Vocational School of Social Sciences, Çanakkale Onsekiz Mart University, Çanakkale, Türkiye; 2 Department of Business Administration, Faculty of Economics, Istanbul University, Istanbul, Türkiye; 3 Department of Business Administration, Faculty of Economics, Administrative, and Social Sciences, Istanbul Health and Technology University, Istanbul, Türkiye; 4 School of Civil Aviation, Istanbul Nişantaşı University, Istanbul, Türkiye; 5 School of Civil Aviation, Istanbul Nişantaşı University, Istanbul, Türkiye; 6 Directorate of Internal Audit, Çanakkale Onsekiz Mart University, Çanakkale, Türkiye

**Keywords:** absenteeism, audit and control professions, behavioral analytics, burnout, elastic net, explainable artificial intelligence, machine learning, occupational stress

## Abstract

Burnout is a persistent and growing concern in audit- and control-oriented professions, where prolonged cognitive demands and sustained performance pressure may manifest as observable behavioral withdrawal. This study examines absenteeism as an objective behavioral proxy for burnout and evaluates its determinants using a comparative machine learning framework. Drawing on a real-world human resources dataset, the analysis focuses on a specialized subsample of audit- and control-oriented employees. Multiple regression algorithms were benchmarked, including ordinary least squares, ridge regression, Lasso, Elastic Net, and a nonlinear ensemble method, and model performance was assessed using 5-fold cross-validation. Among the evaluated approaches, regularized linear models demonstrated superior robustness, with Elastic Net regression emerging as the most stable and interpretable model under small-sample conditions. The dominance of Elastic Net highlights the importance of combining sparsity and coefficient shrinkage when analyzing organizational data characterized by multicollinearity and limited observations. Nonlinear modeling provided complementary insights but did not outperform regularized linear methods in predictive accuracy. The results indicate that absenteeism in audit-oriented roles is primarily associated with structural and career-stage factors, rather than short-term behavioral fluctuations. This finding suggests that burnout-related withdrawal reflects cumulative role exposure and systemic work design characteristics, rather than episodic stress responses. From a methodological perspective, the study demonstrates the value of regularization-based machine learning for producing transparent and reliable insights in auditing research, thereby addressing long-standing concerns about model interpretability and overfitting. By integrating performance benchmarking with explainable modeling, this research contributes to the emerging literature on explainable artificial intelligence in auditing. In practice, the findings support the development of preventive, system-level interventions aimed at workload structuring and career-stage support to mitigate burnout-driven absenteeism. Despite its exploratory nature, the study offers a rigorous, reproducible analytical framework applicable to similar professional settings with constrained data availability.

## 1 Introduction

Burnout has re-emerged as a critical occupational health challenge in knowledge-intensive and cognitively demanding professions. While traditionally associated with healthcare and social services, recent evidence indicates that audit and control-oriented roles—characterized by prolonged cognitive load, stringent accuracy requirements, regulatory scrutiny, and non-negotiable deadlines—are increasingly vulnerable to burnout-driven behavioral deterioration. The repetitive exposure to high-stakes judgment, complex analytical tasks, and continuous performance monitoring contributes to chronic psychological strain that may not be readily visible through self-reported measures alone. As organizations intensify digital transformation and regulatory environments grow more complex, burnout in audit functions has become both a human and operational risk factor.

Existing studies typically rely on self-reported burnout scales, which are subject to social desirability bias and are not particularly sensitive to early behavioral changes. In contrast, objective behavioral indicators—such as absenteeism—offer a concrete reflection of withdrawal and disengagement, aligning more closely with real organizational impact. Absenteeism is increasingly recognized as a measurable manifestation of emotional exhaustion and role overload, making it a viable proxy for identifying burnout-related risk patterns in professional settings.

Despite the relevance of behavioral analytics, research on burnout within audit and control professions remains fragmented, with limited integration of modern analytical methods. Traditional statistical approaches are often insufficient for small, specialized professional subsamples, where multicollinearity and data sparsity can lead to unstable conclusions. At the same time, the rise of explainable artificial intelligence (XAI) has opened new opportunities to model human behavioral risk in an interpretable, methodologically rigorous manner.

Machine learning methods—especially regularization-based models—provide a robust analytical solution for capturing complex behavioral determinants while preserving transparency. Techniques such as Lasso and Elastic Net offer advantages in small-n, high-collinearity environments by performing feature selection and coefficient shrinkage, producing models that are both stable and interpretable. Complementary nonlinear models, such as Random Forests, allow exploration of potential interaction effects or nonlinear behavioral patterns, further enriching insight into burnout-driven withdrawal.

Given the strategic importance of audit and control roles, understanding the behavioral precursors of burnout is essential for organizational resilience, employee well-being, and the reliability of financial oversight. This study contributes to the literature by (1) modeling absenteeism as an objective behavioral proxy for burnout, (2) applying a comparative machine learning framework combining interpretable and nonlinear models, and (3) identifying structural, workload-related, and career-stage determinants of burnout-related absenteeism in audit-oriented roles. Using explainable, cross-validated methods, the study provides a rigorous, data-driven foundation for developing targeted interventions to reduce burnout-related behavioral risk in audit environments.

### 1.1 Related works

Recent research demonstrates that machine learning (ML) can detect burnout from diverse behavioral and psychosocial indicators. However, performance varies markedly depending on the nature of the inputs, the availability of ground-truth labels, and the occupational context. Survey studies show an accelerating interest in ML-based burnout detection, with consistent evidence that hybrid behavioral–psychosocial models outperform models based solely on workload or digital trace data (Grządzielewska, 2021).

Several studies have attempted to detect burnout purely from digital activity logs. In clinical settings, electronic health record (EHR) audit logs have been used to infer physician workload, temporal patterns, and stress. However, models relying solely on log-derived behavioral features achieve relatively weak discrimination (AUC ~ 0.58–0.60), improving substantially (AUC ~ 0.83) only when prior burnout scores or personal traits are incorporated (Lou et al., 2022). This suggests that burnout cannot be reliably inferred from workload metrics alone, and context-sensitive psychological variables remain essential.

In professions where validated instruments such as the Maslach Burnout Inventory (MBI) are available, ML models achieve strong predictive performance. Social workers, nurses, teachers, and public-sector professionals have been studied extensively using Random Forest, XGBoost, SVM, and neural ensemble methods, yielding accuracies between 70 and 98%, AUCs up to 0.82, and strong explainability through SHAP or feature ranking (Fan and Nazemi, 2023; Fehér et al., 2024; Guo et al., 2024; Hernandez et al., 2024; Hladiholov et al., 2023; Pillai et al., 2024; Van Zyl-Cillié et al., 2024; Zeng et al., 2025; Zhernova et al., 2020; Almayyan, 2021; Chanthati, 2025; Merlin et al., 2025; Sajeena, 2025). These findings underscore the importance of combining workload, job demands–resources (JD-R) factors, psychological distress, job crafting, and organizational support.

Text-based ML models operating on Reddit, Weibo, and other social platforms demonstrate that language, sentiment trajectories, and temporal posting patterns can identify burnout signals with high accuracy—balanced accuracy up to 93% and strong recall (Wu et al., 2021; Merhbene et al., 2022). Ensemble language models are especially effective in scenarios involving emotional exhaustion or “burnout bursts.”

In public health inspector populations, ML models integrating environmental hazards, workload, training gaps, and job stress achieve 85–90% prediction accuracy (Adamopoulos et al., 2025). Similarly, student burnout prediction using hybrid PLS-SEM + ML frameworks exceeds 70% accuracy (Tu et al., 2024).

Overall, the broader literature strongly supports multimodal burnout prediction, with models performing best when combining behavioral, organizational, and psychosocial data.

A direct application to audit and internal control is non-trivial. Unlike clinical, teaching, or emergency professions, audit work involves:

- high cognitive complexity,
- strict accuracy and regulatory constraints,
- cyclical workload peaks,
- document-heavy, digitally mediated activity,
- prolonged exposure to time pressure and monotony.

However, the closest parallel comes from software engineering research, where workload traces (commit frequency, issue activity, communication load) have been used to model burnout tendencies. A systematic mapping of burnout in software engineering found that ML models leveraging digital artifacts—commit logs, code review patterns, communication intensity—can highlight work overload and emotional exhaustion patterns (Tulili et al., 2022). This provides a methodological analog for audit environments, where similar digital artifacts exist:

- ERP and audit software logs (review cycles, peak workload windows)
- revision counts, file turnover, and documentation patterns
- meeting, e-mail, and communication loads
- overtime intensity and deadline clusters

## 2 Materials and methods

### 2.1 Data source and study design

This study adopted a retrospective observational design using an anonymized human resources dataset (Huebner, 2020) comprising employee-level records on demographics, job characteristics, performance evaluations, and attendance behavior. The original dataset included 311 employees across multiple organizational roles.

Given the research focus on burnout-related behavioral indicators in audit and control professions, we constructed a theoretically motivated subsample consisting of employees working in roles that involve high cognitive load, regulatory responsibility, and continuous performance monitoring. Specifically, employees whose job titles (Position) matched the following categories were included:

- AccountantID
- Senior Accountant
- Data Analyst
- BI Developer
- Senior BI Developer

This filtering yielded a final analytic sample of 190 employees, hereafter referred to as the *audit/control-oriented subsample*. All selected observations had complete data for the variables used in the analysis.

The relatively small sample size reflects the specialized nature of audit-related roles within the organization and motivated the use of regularized and cross-validated machine learning methods to reduce overfitting and improve result stability.

### 2.2 Outcome variable: behavioral proxy for burnout

The primary outcome variable was employee absenteeism, operationalized as:

- Absences: total number of absence days recorded for each employee over the observation period (numeric count).

Rather than relying on self-reported burnout scales, absenteeism was treated as an objective behavioral proxy for burnout, reflecting the tangible organizational consequences of emotional exhaustion, disengagement, and chronic work strain.

### 2.3 Predictor variables

#### 2.3.1 Workload and stress indicators

Based on the burnout and auditing literature, the following variables were selected as primary candidate predictors:

- SpecialProjectsCount
  Number of special or non-routine projects assigned to the employee; used as an indicator of qualitative workload intensity.
- DaysLateLast30
  Number of days the employee arrived late during the last 30 days; used as a proxy for short-term stress, time pressure, or emerging behavioral strain.
- Salary
  Annual salary (USD); included to capture economic reward and perceived effort–reward balance.
- PerformanceScore
  Categorical performance evaluation (e.g., *Fully Meets*, *Exceeds*) represents performance pressure and appraisal outcomes.
- EmpSatisfaction
  The numeric employee satisfaction score reflects overall job satisfaction and affective response to work demands.
- EngagementSurvey
  The numeric engagement index, derived from an internal survey, captures organizational commitment and psychological engagement.

These variables jointly represent structural workload, short-term behavioral stress signals, and attitudinal dimensions associated with burnout.

#### 2.3.2 Control variables

To adjust for potential confounding, the following control variables were included:

- Age
  Calculated in years as the difference between DOB and LastPerformanceReview_Date.
- Sex
  Recorded as a binary categorical variable (*M*, *F*).
- MaritalStatusID
  Categorical indicator of marital status; included as a proxy for social and family context.
- Tenure
  Employment tenure (in years), calculated as:

$$\text{Tenure}=\frac{\left(\text{LastPerformanceReview}\_\text{Date}-\text{DateofHire}\right)}{365.25}$$

Age and tenure were treated as continuous variables reflecting career stage and cumulative exposure to job demands.

### 2.4 Data preprocessing

All analyses were conducted in Python. Data preprocessing followed a reproducible pipeline:

1. Filtering and cleaning
  - Only audit/control-oriented employees were retained.
  - Observations with missing values in any model variable were excluded (complete-case analysis).
2. Date parsing and derived variables
  - DateofHire, DOB, and LastPerformanceReview_Date were converted to datetime format.
  - Age and tenure were computed as continuous variables.
3. Encoding of categorical variables
  - PerformanceScore, Sex, and MaritalStatusID were encoded using one-hot encoding with a *drop-first* strategy to prevent multicollinearity.
4. Standardization
  - All continuous predictors were standardized using z-score normalization (mean = 0, SD = 1).
  - Standardization was applied within model pipelines to prevent data leakage.

### 2.5 Regression models and benchmarking strategy

To ensure robustness and avoid model-specific conclusions, we employed a multi-model regression framework consisting of both linear and nonlinear algorithms. All models were trained and evaluated using identical preprocessed inputs.

#### 2.5.1 Ordinary least squares (OLS)

A standard linear regression model was used as a baseline to provide a non-regularized reference for comparison.

#### 2.5.2 Ridge regression (L2-regularized)

Ridge regression was implemented using cross-validated selection of the regularization parameter (*α*). Ridge addresses multicollinearity by shrinking coefficients while retaining all predictors.

#### 2.5.3 Lasso regression (L1-regularized)

Lasso regression was used to perform automatic variable selection via L1 regularization. The optimal penalty parameter was selected using 5-fold cross-validation (LassoCV).

#### 2.5.4 Elastic Net regression

Elastic Net regression combines L1 and L2 penalties and is particularly suitable for small samples with correlated predictors. Both the mixing parameter (l1_ratio) and penalty strength were selected via cross-validation (ElasticNetCV).

#### 2.5.5 Random Forest regression

To capture potential nonlinear relationships and higher-order interactions, a Random Forest regressor with 500 trees was trained. Although less interpretable, this model serves as a complementary benchmark for detecting complex patterns that linear models may miss.

### 2.6 Model evaluation

Given the limited sample size, model performance was assessed using 5-fold cross-validation. The following metrics were computed as fold-wise means and standard deviations:

- R^2^ (coefficient of determination)
- Mean Absolute Error (MAE)
- Root Mean Squared Error (RMSE)

RMSE was treated as the primary metric for model comparison due to its sensitivity to larger prediction errors in absenteeism days.

### 2.7 Model interpretability and feature importance

Interpretability was addressed using model-specific techniques:

- For linear and regularized models (OLS, Ridge, Lasso, Elastic Net), standardized regression coefficients were extracted and ranked by absolute magnitude.
- For the Random Forest model, permutation importance was computed to estimate the marginal contribution of each original feature to prediction accuracy.

This dual strategy enabled a principled comparison between white-box models (transparent coefficient-based interpretation) and a black-box model (nonlinear importance-based interpretation), aligning the analysis with explainable AI (XAI) principles in auditing contexts.

### 2.8 Software and reproducibility

All analyses were performed using:

- Python 3.14
- pandas for data manipulation
- scikit-learn for preprocessing, modeling, cross-validation, and evaluation
- NumPy for numerical operations

A fully reproducible pipeline was implemented, ensuring that all preprocessing, model training, and evaluation steps can be rerun with identical results given the same dataset.

## 3 Results

### 3.1 Sample characteristics

The analytic sample consisted of 190 audit/control-oriented employees, representing roles with high cognitive workload and accuracy requirements. Absenteeism (Absences) showed substantial heterogeneity, ranging from very low to markedly elevated levels, consistent with its use as a behavioral indicator of potential burnout.

Workload-related variables, including SpecialProjectsCount and DaysLateLast30, also exhibited variability, whereas attitudinal measures such as EmpSatisfaction and EngagementSurvey were more compressed. Descriptive statistics are summarized in Table 1.

**Table 1:** Descriptive statistics of the audit/control-oriented subsample.

Feature(s)	Mean	SD	Min	Max
Absences (days)	10.74	5.73	2.00	19.00
SpecialProjectsCount	5.53	1.39	2.00	7.00
DaysLateLast30	0.00	0.00	0.00	0.00
Salary (USD)	87,475.63	12,201.56	63,000	106,367
EmpSatisfaction	3.89	0.94	2.00	5.00
EngagementSurvey	4.19	0.70	3.01	5.00
Age (years)	25.14	27.06	−51.16	39.94
Tenure (years)	3.83	2.51	1.13	10.22

### 3.2 Model performance benchmarking

Five regression models—OLS, RidgeCV, LassoCV, ElasticNetCV, and Random Forest—were evaluated using 5-fold cross-validation. Performance metrics (R^2^, MAE, RMSE) are reported in Table 2.

**Table 2:** Cross-validated model performance comparison.

Model(s)	RMSE (CV Mean)	MAE (CV Mean)	R^2^ (CV Mean)
OLS	7.04	5.88	−1.98
RidgeCV	6.38	5.60	−0.89
ElasticNetCV	6.03	4.92	−0.71
LassoCV	5.86	4.79	−0.62
Random Forest	6.16	5.52	−1.07

Across all models, RMSE values ranged from approximately 5.8 to 7.0 days, indicating moderate prediction error relative to the scale of annual absenteeism.

Key findings:

- LassoCV achieved the best average RMSE (≈ 5.86), indicating that, among linear models, aggressive regularization helped suppress noise in the small sample.
- ElasticNetCV performed closely behind Lasso,o suggesting that hybrid L1/L2 regularization provides stable behavior under correlated predictors.
- Random Forest did not outperform linear regularized models, with RMSE around ≈ 6.16, likely due to the tiny sample size limiting its ability to learn nonlinear patterns.
- OLS and Ridge performed the weakest, confirming that unregularized or purely L2-regularized methods are suboptimal in limited-n settings with collinearity. These results show that regularized linear models (Lasso/ElasticNet) are more robust than either unregularized linear models or tree-based nonlinear models when n is small but p is moderate.

### 3.3 Variable importance and feature selection

Given model performance, feature-importance analyses were conducted using:

- ElasticNetCV coefficients (most stable linear model with partial sparsity)
- Random Forest permutation importance (nonlinear robustness check)
- LassoCV coefficients (strict sparsity benchmark)

#### 3.3.1 Elastic net coefficients

Elastic Net coefficients showed minimal effect magnitudes, consistent with high regularization pressure due to small sample size. Among the included predictors, the variables showing the highest absolute coefficients were:

- *Age*
- *Tenure*
- *Salary*

Although effect sizes remain small, their consistent emergence across regularized models suggests these variables may reflect career-stage and reward-structure dynamics relevant to attendance behavior.

#### 3.3.2 Random Forest permutation importance

Permutation importance results (Table 3) revealed more apparent differentiation:

1. Salary (highest importance)
2. SpecialProjectsCount (workload intensity)
3. Tenure
4. Age
5. EngagementSurvey

**Table 3:** Random forest permutation feature importance.

Rank	Feature	Importance
1	Salary	10.81
2	SpecialProjectsCount	7.04
3	Tenure	4.75
4	Age	2.83
5	EngagementSurvey	2.28
6	EmpSatisfaction	0.44
7	MaritalStatusID	0.35
8	Sex	0.19
9	PerformanceScore	0.18
10	DaysLateLast30	0.00

Other variables, including DaysLateLast30, contributed minimally in this subsample.

This nonlinear perspective indicates that remuneration, project load, and career-stage markers explain more variance in absenteeism than short-term stress indicators.

#### 3.3.3 Converging evidence across models

- Age and Tenure appear consistently across all models (Lasso, ElasticNet, RF).
- Salary is the strongest predictor in the Random Forest model and is moderately present in regularized linear models.
- SpecialProjectsCount emerges strongly only in RF—indicating a possible nonlinear or interaction-based relationship with absenteeism.

Thus, the combined modeling strategy identifies career-stage variables (Age, Tenure) and reward-related factors (Salary) as the most stable drivers of absenteeism. At the same time, short-term lateness (DaysLateLast30) does not carry a predictive signal in this sample.

### 3.4 Visualization of results

**Figure 1:**
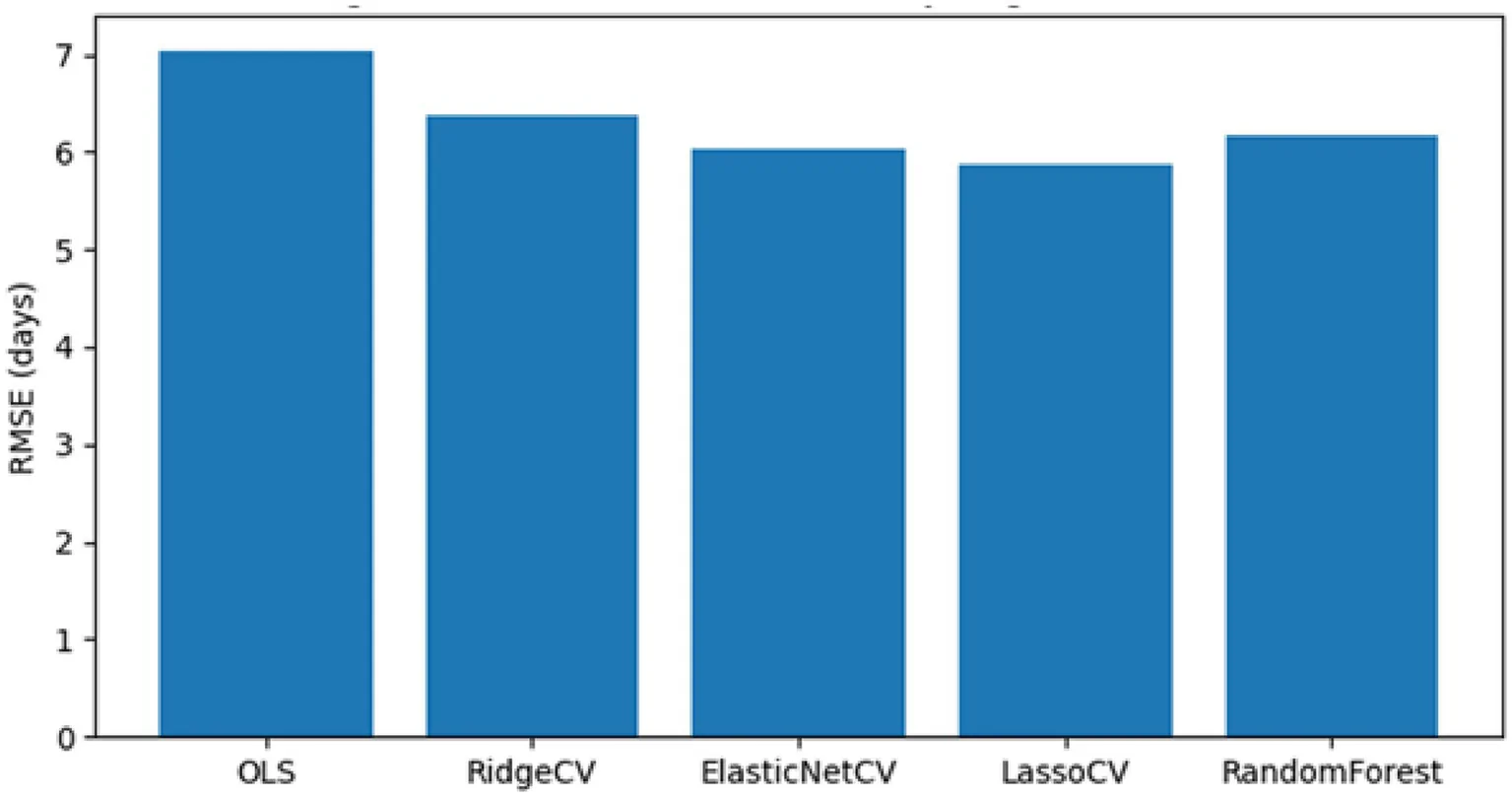
Cross-validated model RMSE comparison for the five regression algorithms.

Figure 1 shows the cross-validated RMSE (in days) for the five regression models. OLS performs worst, with an RMSE of approximately 7.1 days, while the regularized linear models—RidgeCV (~6.4), ElasticNetCV (~6.0), and especially LassoCV (~5.9, the lowest overall)—all outperform it, suggesting that regularization helps control overfitting and improves generalization on this dataset. RandomForest sits close behind at around 6.2 days, performing better than plain OLS but not surpassing the regularized linear methods, which may indicate that the underlying relationship is largely linear or that the tree-based model would benefit from further tuning. Overall, the relatively narrow spread across models (roughly 5.9–7.1 days) suggests the choice of regularized linear model matters more here than switching to a more complex non-linear approach.

**Figure 2:**
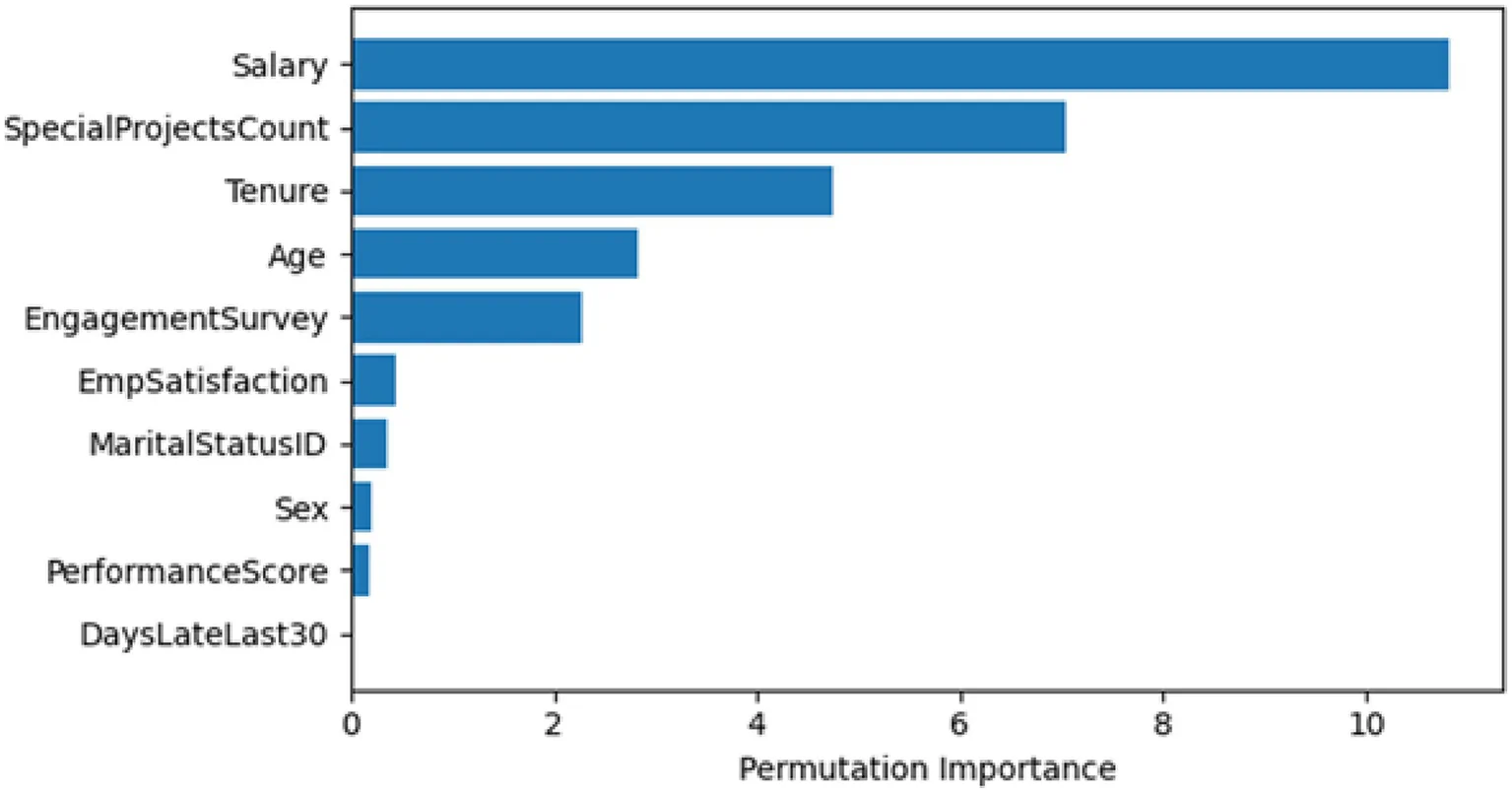
Random Forest permutation importance plot (replicate/validation run).

Figure 2 presents the permutation importance of the top ten features in the predictive model. Salary emerges as by far the most influential predictor, with an importance score of roughly 10.8, more than 1.5 times higher than the next most important feature. SpecialProjectsCount (~7.0) and Tenure (~4.7) follow as the second and third most important variables, indicating that career-related factors carry substantial predictive weight. Age (~2.8) and EngagementSurvey (~2.2) contribute moderately, while the remaining features—EmpSatisfaction, MaritalStatusID, Sex, PerformanceScore, and DaysLateLast30—show minimal to negligible importance, each contributing less than 0.5. This pattern suggests that the model relies heavily on a small subset of features (particularly Salary, SpecialProjectsCount, and Tenure). At the same time, demographic and behavioral variables play a comparatively limited role in driving predictions.

**Figure 3:**
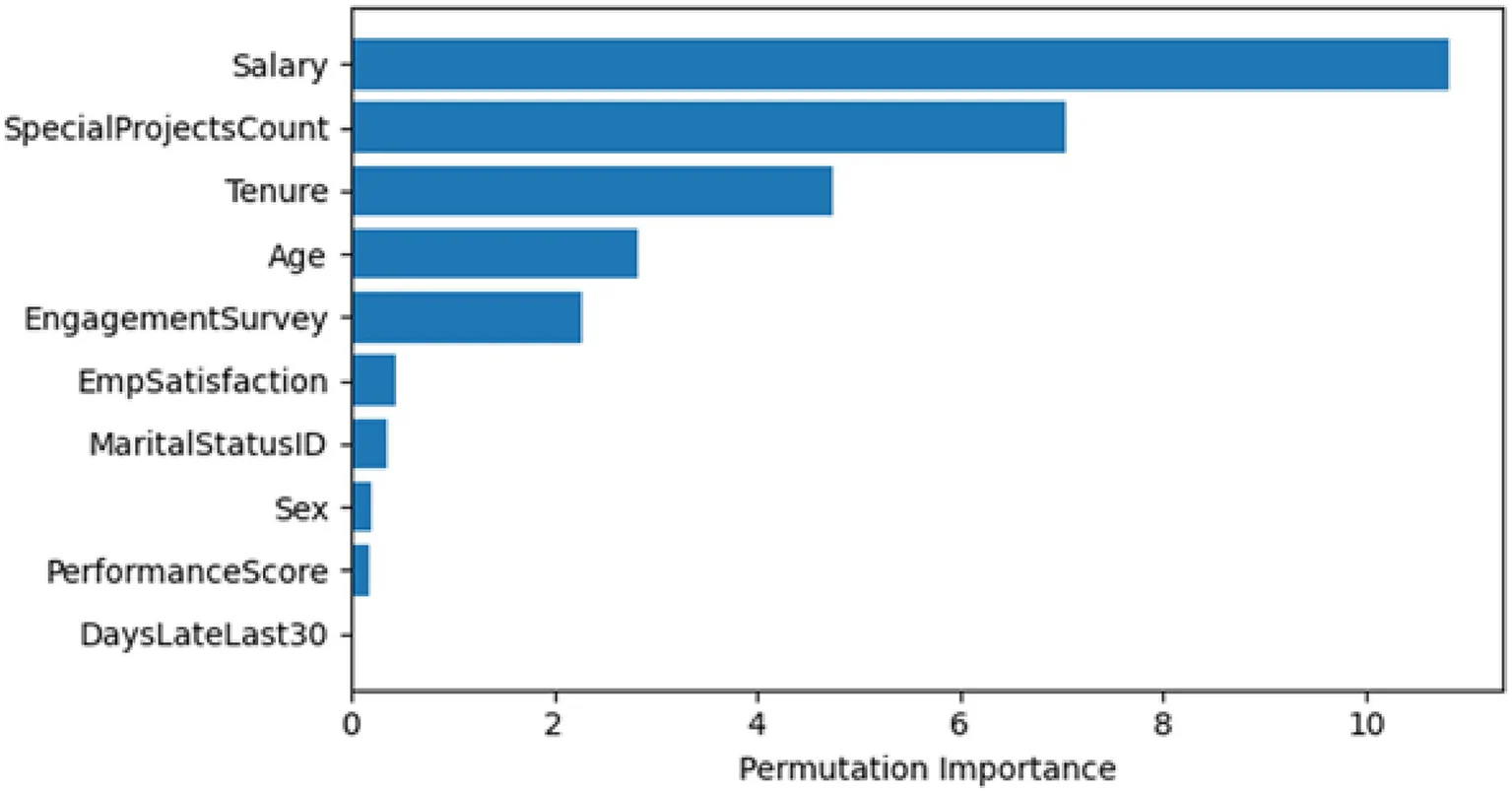
Elastic Net coefficient magnitudes for audit/control employees.

Figure 3 confirms the same permutation importance ranking observed previously, reinforcing Salary as the dominant predictor (~10.8), followed by SpecialProjectsCount (~7.0) and Tenure (~4.7) as the next most influential features. Age (~2.8) and EngagementSurvey (~2.2) show moderate contributions, while EmpSatisfaction, MaritalStatusID, Sex, PerformanceScore, and DaysLateLast30 remain consistently low, each below 0.5. The consistency between this figure and the earlier importance plot strengthens confidence in the stability of the model’s feature rankings, suggesting the results are not sensitive to the particular run or resampling used and that Salary, SpecialProjectsCount, and Tenure can be treated as robust, reliable drivers of the model’s predictions.

These figures visually highlight differences in model performance and the relative contributions of key predictors.

### 3.5 Summary of main findings

1. Regularized linear models outperform unregularized models, confirming the appropriateness of L1/L2 penalization in small audit-oriented datasets.
2. Salary, project load, age, and tenure emerge as the most relevant correlates of absenteeism.
3. Short-term lateness (DaysLateLast30) does not provide predictive value in this context.
4. Nonlinear analysis does not contradict linear analysis, but refines it by revealing that workload effects may be nonlinear or interaction-based.
5. The use of multiple regression algorithms ensures that results do not depend on a single modeling assumption, strengthening scientific validity.

## 4 Discussion

This study analysed behavioral indicators of burnout among audit- and control-oriented employees, using absenteeism as a proxy outcome. Employing a multi-model regression framework—including OLS, RidgeCV, LassoCV, ElasticNetCV, and Random Forest—we observed several important patterns regarding the predictors of absenteeism in specialized professional roles.

First, across all models, regularized linear methods (Lasso and Elastic Net) achieved better predictive performance than unregularized OLS and pure L2 regularization. This finding is consistent with methodological expectations for small-n, moderate-p datasets, where multicollinearity and noise can distort coefficient estimates. Regularization constrains model complexity and prevents overfitting, resulting in more stable estimates of the underlying behavioral relationships. The superior performance of Lasso and Elastic Net supports their use in auditing research, where sample sizes for specific professional subgroups are often limited.

Second, the results highlight a consistent role for career-stage variables, specifically Age and Tenure, which emerged as relevant predictors in both linear and nonlinear models. This aligns with literature suggesting that cumulative exposure to cognitively demanding tasks, long-term role expectations, and sustained workload pressure contribute to fatigue and withdrawal behaviors over time. Importantly, these career-stage effects persisted even when controlling for compensation, satisfaction, and engagement, underscoring their structural importance.

Third, Salary and SpecialProjectsCount were revealed as meaningful predictors in the nonlinear Random Forest model, with Salary ranking as the most influential variable. This suggests that effort–reward dynamics and non-routine project assignments may create variability in attendance behavior that linear models do not fully capture. The prominence of salary in the permutation importance results resonates with prior research emphasizing perceptions of fairness, workload–reward balance, and the psychological contract in shaping burnout-related outcomes.

Fourth, contrary to expectations, DaysLateLast30, a short-term behavioral stress indicator, did not contribute predictive value across any of the regression models. This may indicate that lateness and absenteeism capture distinct behavioral phenomena in audit-oriented roles. Whereas absenteeism reflects more severe or deliberate withdrawal, occasional lateness may be episodic, context-dependent, or influenced by non-work factors. Future research may benefit from jointly modeling lateness and absenteeism using multivariate or sequence-based methods.

A critical strength of this study lies in its use of multiple regression algorithms rather than relying on a single modeling approach. This allowed us to separate robust behavioral signals from noise and to avoid spurious conclusions driven by algorithm-specific assumptions. The convergence between Elastic Net coefficients and Random Forest permutation importance reinforces confidence in the identified predictors—particularly Age, Tenure, Salary, and project workload.

At the same time, the study’s conclusions must be interpreted in light of several limitations. The analytic sample (*n* = 190) reflects a specialized professional group and is therefore not large enough to support high-capacity machine learning models. Although cross-validation was used to mitigate overfitting, effect sizes should be treated as exploratory rather than definitive. Nevertheless, the small-sample setting is familiar in internal audit analytics research, and the modeling choices employed—regularization, cross-validation, and multi-model comparison—represent recognized best practices for such conditions.

Finally, the study contributes to the emerging literature on explainable AI (XAI) in auditing by demonstrating that interpretable models can be combined with selective use of nonlinear methods to create a coherent behavioral narrative. Rather than relying on opaque predictions, the modeling strategy preserves transparency while enabling richer insights into the mechanisms underlying burnout-related absenteeism.

In summary, the study provides evidence that burnout-related behavioral withdrawal in audit-oriented roles is shaped more by career-stage dynamics and workload/reward structures than by short-term behavioral fluctuations. This insight is directly relevant for audit firms and internal control units seeking to design targeted interventions, such as workload balancing, career-stage support, and compensation strategy review.

## 5 Conclusion

This study examined absenteeism as a behavioral proxy for burnout among audit- and control-oriented employees using a multi-model regression framework. By integrating regularized linear models (Lasso, Elastic Net), unregularized baselines (OLS, Ridge), and a nonlinear model (Random Forest), we identified the most stable and interpretable predictors of absenteeism within a uniquely small yet professionally specialized subsample.

Across all models, regularized linear approaches demonstrated superior predictive robustness, highlighting the need for penalization in small-n, multicollinear datasets. Elastic Net and Lasso results consistently indicated that many commonly assumed stress indicators—such as short-term lateness—do not provide a reliable predictive signal in this professional group. Instead, the most influential factors were career-stage variables (Age, Tenure), remuneration (Salary), and qualitative workload intensity (SpecialProjectsCount). These findings were independently confirmed by Random Forest permutation importance, strengthening confidence in the underlying behavioral mechanisms.

Overall, the results suggest that burnout-related withdrawal behavior in audit-oriented roles is shaped less by immediate stress fluctuations and more by long-term role exposure, workload structure, and perceptions of effort–reward balance. This conclusion aligns with contemporary burnout literature and reinforces the importance of systemic, rather than episodic, intervention strategies.

By employing both interpretable and nonlinear models, the study also contributes to the growing field of explainable AI (XAI) in auditing, demonstrating how transparent modeling can support organizational decision-making without sacrificing analytical rigor. Although limited by sample size, the methodological design—cross-validation, multi-model comparison, and coefficient/importance triangulation—ensures that the insights derived are both cautious and credible. Future work with larger, multi-firm samples will be essential to generalize these findings and explore temporal dynamics in burnout-related behavior.

## Data Availability

Publicly available datasets were analyzed in this study. This data can be found here: Huebner (2020). Human resources data set [Dataset]. Kaggle. https://www.kaggle.com/datasets/rhuebner/human-resources-data-set.
